# A new species of knob-scaled lizard (Xenosauridae, *Xenosaurus*) from the Sierra Madre Oriental of Puebla, Mexico

**DOI:** 10.3897/zookeys.737.15095

**Published:** 2018-02-15

**Authors:** Adrián Nieto-Montes de Oca, Helder Sánchez-Vega, Itzel Durán-Fuentes

**Affiliations:** 1 Laboratorio de Herpetología and Museo de Zoología Alfonso L. Herrera, Departamento de Biología Evolutiva, Facultad de Ciencias, Universidad Nacional Autónoma de México, Cd. Universitaria, Del. Coyoacán, Ciudad de México, C. P. 04510, México

**Keywords:** Mexico, new species, Puebla, Sierra Madre Oriental, Xenosauridae, *Xenosaurus*, *Xenosaurus tzacualtipantecus* clade

## Abstract

A new species of *Xenosaurus* in the *X. tzacualtipantecus* clade is described from the Sierra Madre Oriental of northern Puebla, Mexico. The new species differs from all of its congeners in possessing a unique combination of characters. The new species appears to be allopatric and fills in the geographic gap between the geographic distributions of *X. tzacualtipantecus* and the species in the *newmanorum* clade to the north and northwest and those of the species in the *grandis* and *rackhami* clades to the south and southeast. The new species occurs between approximately 880 m and 1470 m of elevation, and appears to be restricted to cloud forest, which has been replaced by coffee plantations in many areas. An updated key to the species of *Xenosaurus* is provided.

## Abstract (es)

Se describe una nueva especie de *Xenosaurus* del clado *X. tzacualtipantecus* de la Sierra Madre Oriental del norte de Puebla, México. La nueva especie difiere de todos sus congéneres por poseer una combinación única de caracteres. La nueva especie parece ser alopátrica y llena el hueco geográfico entre las distribuciones geográficas de *X. tzacualtipantecus* y las especies del clado *newmanorum* hacia el norte y noroeste, y aquellas de las especies de los clados *grandis* y *rackhami* hacia el sur y sureste. La nueva especie se ha encontrado en elevaciones entre aproximadamente 880 m y 1470 m y parece estar restringida al bosque mesófilo de montaña, el cual ha sido reemplazado por cafetales en muchas áreas. También se ofrece una clave actualizada para las especies de *Xenosaurus*.

## Introduction

Lizards of the Middle American genus *Xenosaurus* Peters, 1861 occur from southwestern Tamaulipas and extreme southeastern Guerrero, on the Caribbean and Pacific versants of Mexico, respectively, south and east to Alta Verapaz, Guatemala (King and Thompson 1968; Smith and Iverson 1993; Ballinger et al. 2000; Pérez-Ramos et al. 2000; Nieto-Montes de Oca et al. 2001; Canseco-Márquez 2005; Zamora-Abrego 2009; Nieto-Montes de Oca et al. 2013, 2017). Species of *Xenosaurus* occur in a considerable elevational range (ca. 300–2360 m; King and Thompson 1968; Smith and Iverson 1993; Ballinger et al. 2000) and are inhabitants of a wide variety of habitats, ranging from xerophytic tropical scrub to cloud forest and tropical rain forest (King and Thompson 1968; Ballinger et al. 2000).

Twelve species of *Xenosaurus* have been described: *X. grandis* (Gray, 1856), *X. rackhami* Stuart, 1941, *X. newmanorum* Taylor, 1949, *X. sanmartinensis* Werler & Shannon, 1961, *X. arboreus* Lynch & Smith, 1965, *X. agrenon* King & Thompson, 1968, *X. platyceps* King & Thompson, 1968, *X. rectocollaris* Smith & Iverson, 1993, *X. penai* Pérez-Ramos, Saldaña de la Riva & Campbell, 2000, *X. phalaroanthereon* Nieto-Montes de Oca, Campbell & Flores-Villela, 2001, *X. tzacualtipantecus* Woolrich-Piña & Smith, 2012, and *X. mendozai* Nieto-Montes de Oca, García-Vázquez, Zúñiga-Vega & Schmidt-Ballardo, 2013. The taxonomic history of the genus and the geographic distribution of its species have been recently summarized by Nieto-Montes de Oca et al. (2013) and Nieto-Montes de Oca et al. (2017, Fig. 2).

In addition to the above species, a recent phylogenetic analysis of the genus *Xenosaurus* based on RADseq data (Nieto-Montes de Oca et al. 2017) provided evidence that its diversity has been substantially underestimated, and that several species remain to be described. Given the usually restricted distributions of these species and the destruction of their habitats, such description is a pressing matter. In this study, we describe the undescribed species from the Huehuetla region, Puebla, referred therein. Because the number of described species of *Xenosaurus* has nearly doubled since the only key to its species was published (King and Thompson 1968), an updated key to the species in the genus is also provided.

## Materials and methods

Field work conducted in the Sierra Madre Oriental of Puebla during the last two decades by herpetologists at the Museo de Zoología Alfonso L. Herrera, Facultad de Ciencias, Universidad Nacional Autónoma de México (MZFC) led to the collection of a total of 10 specimens of *Xenosaurus*, which were fixed in 10% buffered formalin, stored in 70% ethanol, and deposited in the MZFC. These specimens were compared with specimens of all of the described species of *Xenosaurus*, including the type series of *X. agrenon*, *X. arboreus*, *X. mendozai*, *X. penai*, and *X. phalaroanthereon*. A list of the specimens examined is provided in the Suppl. material 1. Institutional abbreviations for museums and collections follow Sabaj (2016), except for IBH-LT (Estación de Biología Tropical “Los Tuxtlas,” Instituto de Biología, Universidad Nacional Autónoma de México, originally in Veracruz, Mexico; now housed in CNAR).

Nomenclature of scales follows King and Thompson (1968), Smith and Iverson (1993), and Nieto-Montes de Oca et al. (2001). Scale counts were performed using a dissecting microscope. Measurements were taken with calipers to the nearest 0.1 mm. Head length and snout length were measured from the tip of the snout to the anterior margin of the ear and from the tip of the snout to the anterior margin of the orbit, respectively. In the case of characters examined on both the left and right sides of each specimen, the corresponding conditions are reported in this order, separated by a slash.

## Results

### Xenosaurus fractus sp. n.

Taxon classificationAnimaliaSquamataXenosauridae

http://zoobank.org/902E3E2C-8A7C-4D51-9AEB-A7BFD9A17B07

Figs 1 , 2 , 3 , 4

#### Holotype

**Figure 1.**
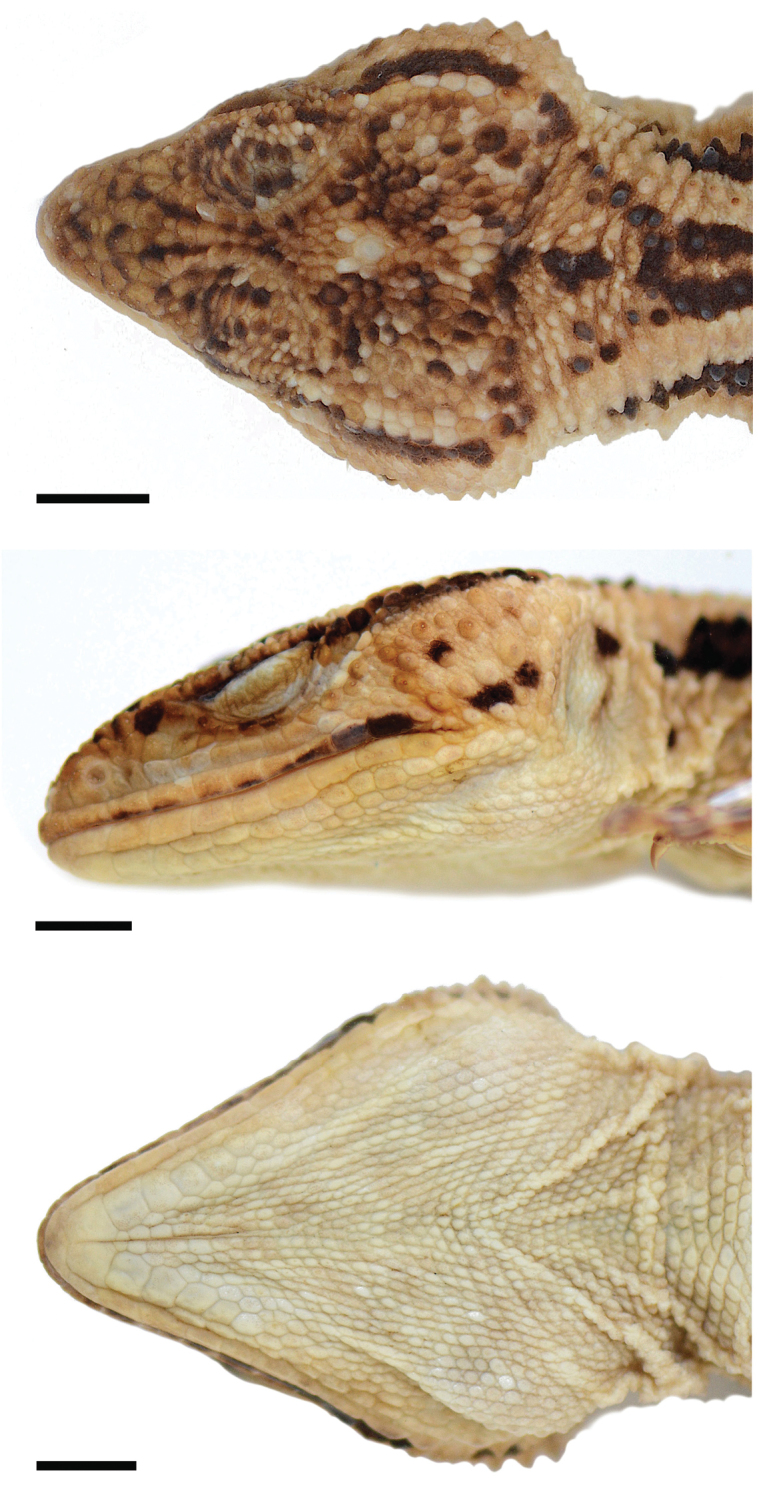
*Xenosaurus fractus*, holotype (MZFC 32179). Head scales in (top) dorsal view; (middle) left lateral view; and (bottom) ventral view. Head length = 27.6 mm. Scale bars: 5 mm.

**Figure 2.**
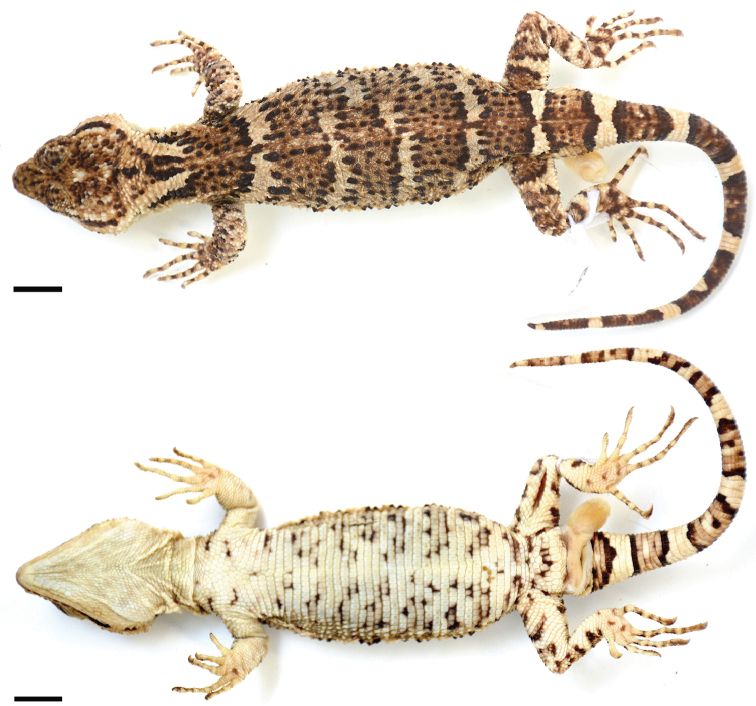
*Xenosaurus fractus*, holotype (MZFC 32179). Color pattern in (top) dorsal view; (bottom) ventral view. Snout-vent length = 115.3 mm. Scale bars: 10 mm.

(Figs 1–2). An adult male (MZFC 32179, field number EPR 1375) from Puebla, Municipality of Huehuetla, approximately 0.7 km N Chilocoyo del Carmen, 20.0862°N, 97.6530°W, 980 m elevation; rocky outcrop between coffee plantations and cloud forest patches; rock crevice; collected by Edmundo Pérez Ramos, Itzel Durán Fuentes, and Walter Schmidt Ballardo on 8 June 2003.

#### Paratypes

(Fig. 4). Nine; all from Puebla: six topotypes (MZFC 32180–85); one from the Municipality of Huehuetla, Chilocoyo del Carmen, 20.0802°N, 97.6549°W, 880 m (MZFC 9579); one from the Municipality of Huehuetla, “Huehuetla,” 20.1108°N, 97.6529°W, 950 m (approximately 2.8 km W Huehuetla) (MZFC 32187); and one from the Municipality of Xochitlán, “ribera del rio Apulco, near Zacapoaxtla” (approximately 5.6 km SSE Xochitlán), 19.9225°N, 97.6046°W, 1470 m (MZFC 32186).

#### Diagnosis

(Suppl. material 2). *Xenosaurus fractus* may be distinguished from all of the other species of *Xenosaurus*, except *X. tzacualtipantecus*, by lacking a continuous dark crossband on the nape, or collar; and having instead a funnel-shaped mark on the nape formed by the dorsal color pattern of the head extending posteriorly, gradually narrowing on the nape (while bordered by the posterior extension of a narrow, dark brown stripe on the canthus temporalis, the posterior extension of a broad, cream subocular stripe, and a broad, black stripe on each side), to the first brown crossband on the trunk (posterior extensions of the subocular stripes remaining narrowly separated medially at their posterior end), versus a mainly uninterrupted dark crossband on the nape enclosed anteriorly by the pale subocular stripes, which extend medially onto the nape producing a pale crossband (often interrupted medially) similar to those on the trunk, and a pale crossband in the scapular region separating the dark crossband on the nape from the first dark crossband on the trunk in the other species.

*Xenosaurus fractus* may be distinguished from *X. tzacualtipantecus* by having, on average, more subdigital scales on the fourth toe (26–34, x = 29.9, *n* = 10; versus 23–28, x = 25.6, *n* = 8, in *X. tzacualtipantecus*) and dorsal surface of the limbs barred (black-edged, cream bars on mid upper arm, forearm, thigh, and shank; versus limbs usually not barred in *X. tzacualtipantecus* [upper arm, forearm, thigh, and shank specked with black; specks usually not forming a distinct pattern; coalescing into narrow lines, bordering ill-defined bars and showing a tendency to anastomose, on thigh and shank in one specimen; *n* = 8]).

#### Description of holotype

(Figs 1–2). Adult male (MZFC 32179), snout-vent length (SVL) = 115.3 mm, moderately robust body, and limbs and tail intermediate in length (shank length = 17.1 mm [14.8 % SVL]; tail length = 106.2 mm [92.1 % SVL]).

Head intermediate in size (length = 27.6 mm [23.9 % SVL], maximum width = 23.8 mm [20.6 % SVL]), moderately flattened (maximum height = 13.6 mm [49.4 % head length]), broadly triangular in dorsal view (snout length = 10.0 mm, 36.3 % head length). Scales on dorsal surface of head differentiated in size and relief; those on snout moderately enlarged, slightly irregular, protruding, occasionally bluntly keeled; scales of supraorbital semicircles and scales between them also moderately enlarged and protruding, but most of them bluntly keeled anteriorly; gradually becoming smaller, lower, not keeled posteriorly; scales of supraocular region smooth; moderately enlarged, flat, on center of region; small, slightly bulging, around center (especially on anterior end); scales of parietal region small to mid-sized, flat to slightly elevated, smooth; those on canthi temporales bluntly keeled.

Rostral moderately large, approximately one-third as high as wide, its dorsal margin straight; split vertically into two scales by sulcus extending from its dorsal margin; left scale approximately one-third as wide as right one; combined scales as wide as mental; dorsal fourth of right scale divided into three by two short sulci extending ventrally from its dorsal margin. Three medium-sized postrostrals; medial postrostral asymmetrical, its left margin much higher than right one, its dorsal margin oblique; lateral postrostral in contact with nasal on each side; left postrostral triangular, approx. as wide as high; right postrostral rectangular, approximately 1.5 times as wide as high. Scales on remaining dorsal surface of snout undifferentiated; minimum count of five scales between supranasals at level of nostrils; minimum count of five scales across snout in front of anterior end of supraorbital semicircles. Supraorbital semicircles separated along midline by one scale row; minimum count of scales between supraorbital semicircles and interparietal 3/3. Interparietal small (length = 1.55 mm), approximately twice as long as scales around it. Scales of supraocular region arranged in a main row of 5 / 5 moderately enlarged, moderately wider than long, flat scales roughly parallel to supraorbital semicircles, and mostly distinctly smaller scales around latter row (those in lateral contact with fourth and fifth scales in right row only slightly smaller than them); widest scales in main row approximately 2.5–3.0 times as wide as long; remaining scales narrower, yet still wider than long; scales of main row separated at level of mid orbit from supraorbital semicircles by one row of small and medium-sized scales, and from superciliaries by one short row of medium-sized scales and two rows of small scales on left side, and by 2–3 ill-defined rows of small scales on right side. Superciliaries 15/14, small, roughly square to slightly longer than wide. Parietal scales small to mid-sized, minimum count of scales across parietal area approximately 22.

Nasal scale moderately large, 0.8 times as high as wide, completely encircling nostril at its posterodorsal corner; one medium-sized supranasal; two postnasals (one small, dorsal one and one medium-sized, ventral one); posterodorsal corner of nasal bordered by one small/mid-sized obliquely oriented scale. Canthus rostralis rounded; not differentiated, canthal scales separating scales on dorsal and lateral surfaces of snout; scales on loreal region small to medium-sized, irregular, elevated, roughly arranged in two horizontal rows, not keeled except for those of lorilabial scale row. Fifteen/twelve scales around orbit between canthus rostralis and canthus temporalis (preocular-subocular-postocular series); uppermost two preoculars large, protruding; remaining scales in series medium-sized, also protruding, keeled; their keels forming a well-defined subocular-postocular ridge. Lorilabial scales large, approx. twice as high as suboculars and slightly higher than supralabials below level of mid-eye. Zygomatic ridge poorly developed, composed of medium-sized, moderately protruding scales. Ventral portion of suboculars and dorsal portion of lorilabials sunken, forming a groove extending posteriorly from loreal region below orbit, then curving dorsally behind orbit between postocular and zygomatic ridges; ridges separated from each other by one continuous row of small scales. Canthus temporalis rounded; outermost row of parietal scales bordered laterally, posterior to zygomatic ridge, by some granular scales and then by one row of small, low, blunt conical tubercles, slightly larger posteriorly. One row of medium-sized, roughly conical tubercles (tapering from roughly circular to vertically oval bases) bordering tympanum posteriorly; small to medium-sized (occasionally large), rounded, conical tubercles scattered on most of temporal area, separated by 1–3 rows of granular scales from each other. Tympanum large, vertically oval (width = 3.1 mm, height = 5.7 mm), its height 20.7 % of head length; covered with a thick membrane of small, granular scales. Supralabials 11/10; anterior ones smooth; last 2/3 slightly elevated, with low crest. Infralabials 9/10, slightly convex, bluntly keeled; gradually becoming more elevated and distinctly keeled towards posterior end of row.

Mental large, slightly more than twice as wide than long, bordered posteriorly by two large chinshields between infralabials; each chinshield followed posteriorly by five gradually smaller chinshields, forming one chinshield row extending posterolaterally parallel to infralabial row. Second chinshields separated medially by two rows of small scales. Labiomental row extending anteriorly to approximately middle of third infralabial. Scales in labiomental row small, keeled, gradually becoming slightly larger towards posterior end of row; last scale in row much larger, higher, tubercular, extending to midpoint between end of mouth and tympanum. Rest of throat and gular region covered by small gular scales. Gular fold moderately well developed.

Scales on dorsal surface of body differentiated into small to mid-sized, rounded, convex tubercles and minute granules between tubercles; tubercles roughly uniformly distributed; most tubercles separated from each other by distances slightly smaller to slightly larger than their own diameter; tubercles on trunk roughly arranged in transverse rows; tubercles smaller, lower, flattened on middorsal region (smallest along midline); gradually becoming higher, conical, toward flanks; approximately 30 paravertebral tubercles between levels of axilla and groin. Poorly developed lateral fold close to ventral scales. Ventral scales on chest arranged in slightly oblique rows; gradually becoming arranged in transverse rows beyond level of axilla. Scales on chest and venter rectangular, slightly longer than wide; some with small, circular central depression; transverse scale rows along midline between levels of axilla and groin approximately 34; scales in a transverse row at level of midbody 23. About four transverse rows of enlarged, smooth, subsquare or subrectangular precloacal scales; enlarged scales in posteriormost precloacal row eight.

Scales on dorsal surface of limbs differentiated into small, rounded, conical tubercles and minute granules between tubercles; tubercles on arms usually pointed, separated from each other by one granular scale row, slightly more widely separated on posterior surface of forearm; tubercles on legs usually pointed on thighs (point often off center), bluntly pointed on shank; usually separated from each other by 1–2 granular scale rows; slightly more widely separated on posterior surface of thighs. Subdigital lamellae on fourth toe 29/31; length of fourth toe 17.0/17.0 mm.

Tail circular in cross section, moderately wide at base; scales on dorsal and lateral surfaces of anterior end arranged in transverse rows of small, rounded, slightly convex tubercles separated by granular scales, and scales on ventral surface small, square, juxtaposed; scales on all surfaces gradually becoming rectangular, longer than wide, juxtaposed posteriorly. Simple rows of scales, composed of scales on ventral surface gradually narrowing on lateral and dorsal surfaces, alternating with scale rows composed of scales on ventral surface extending dorsally, bifurcating on sides, into two narrow rows encircling tail on dorsal surface (i.e., scales of adjacent rows similar in length on either dorsal or ventral surfaces; combined length of two adjacent rows on ventral surface approximately equal to combined length of three adjacent rows on dorsal surface).

Color in preservative (Fig. 2). Dorsal background color of head pale brown, speckled with dark brown and cream; lateral background color cream, except for dark brown uppermost preocular; narrow, dark brown, fragmented line on upper lip (wider on last three supralabials); narrow, dark brown stripe on canthus temporalis, and few dark brown spots on temporal area, including a spot on posteroventral corner of temporal area; ventral surface immaculate cream.

Background color of body, limbs, and tail cream. Dorsal pattern of body composed of a brown, middorsal funnel-shaped mark on nape and five brown crossbands on body; funnel-shaped mark formed by dorsal color pattern of head extending posteriorly, gradually narrowing on nape (bordered by posterior extension of narrow, dark brown stripe on canthus temporalis, posterior extension of broad, cream subocular stripe, and a broad, black stripe on each side) to first brown crossband on trunk nearly at level of anterior insertion of arms (posterior extensions of subocular stripes approaching each other posteriorly but remaining narrowly separated medially at their posterior end).

Brown crossbands on body broad, with black tubercles along their anterior and posterior borders and also scattered between them; separated by cream, transverse, narrower interspaces except for second, third, and fourth bands partially fused on right side. Limbs with narrow, cream transverse bars bordered laterally by black narrow lines on middle of upper arms, forearms, thighs, and shanks; narrower, poorly defined, additional black-edged cream transverse bar on distal end of thighs and an irregular pale spot on knee; remaining dorsal surface of limbs speckled with black and cream; digits with alternating cream and brown rings. Ventral surface immaculate on most of neck; with irregular, transverse, fragmented narrow bars on sides of chest and abdomen, precloacal area, and outer borders of legs.

Tail with eight transverse, somewhat irregular, black-edged brown rings; posterior end dark brown; sixth and seventh rings partially fused; rings speckled with dark brown and rarely white dorsally; anterior rings with cream midventral spot on their middle.

#### Variations.

Here variations found in scalation in the nine paratypes and in color pattern in eight is reported upon (see below). Rostral split vertically into two or three scales by sulci extending ventrally from its dorsal margin in three specimens: split into two by sulcus on left side, in one specimen; split into three by one sulcus on each side, in two specimens; rostral with short sulci in remaining six specimens. Lateral postrostrals 2/1 in one specimen, 1/1 in remaining eight. Enlarged supraoculars in main supraocular rows approximately 4/4 in five specimens, 4/3 in two (first two scales split into two on each side in one), 3/3 in two (one scale split into two on each side in one). Widest supraoculars in main supraocular rows approximately 1.5–2.0 times as wide as long in two specimens, 2.0–2.5 times as wide as long in three, 2.5–3.0 times as wide as long in four. Superciliaries on right side 10–12, x = 11.2; parietal scales across parietal region 18–21, x = 19.8.

Postocular and zygomatic ridges in contact only at their ventral ends in one specimen; along their ventral halves in two; along their ventral two thirds in one; throughout their length, except separated by one small scale, in two; in contact throughout their length in three. Canthus temporalis weakly angular anteriorly, gradually becoming rounded on its posterior half to two thirds, in six specimens; moderately angular to angular throughout its length in three. Supralabials on right side 9–13, x = 11.2; infralabials on right side 9–11, x = 9.9.

Labiomental row extending anteriorly to between levels of mid second and mid third infralabials in all paratypes; extending posteriorly to midpoint between mouth and tympanum (chinshields gradually becoming smaller posteriorly, merging with gular scales between levels of posterior end of fourth and mid sixth infralabials) on both sides in six specimens (MZFC 32180, 32182–84, 32186–87) and on right side in one (MZFC 9579); chinshields gradually becoming smaller posteriorly, bordering labiomental row medially and then posteriorly, extending parallel to infralabial row to midpoint between mouth and tympanum on both sides in two specimens (MZFC 32181, 32185) and on left side in one (MZFC 9579).

Tubercles in a paravertebral row between levels of axilla and groin 27–37, x = 30.7. Midventral scales between levels of axilla and groin 33–35, x = 33.9; longitudinal rows of ventral scales at level of midbody 22–25, x = 22.4. Transverse rows of enlarged scales on precloacal region 6–8, x = 7; scales in posteriormost transverse row of precloacal scales 6–9, x = 7.7. Subdigital lamellae on fourth toe 26–34, x = 29.9.

**Figure 3.**
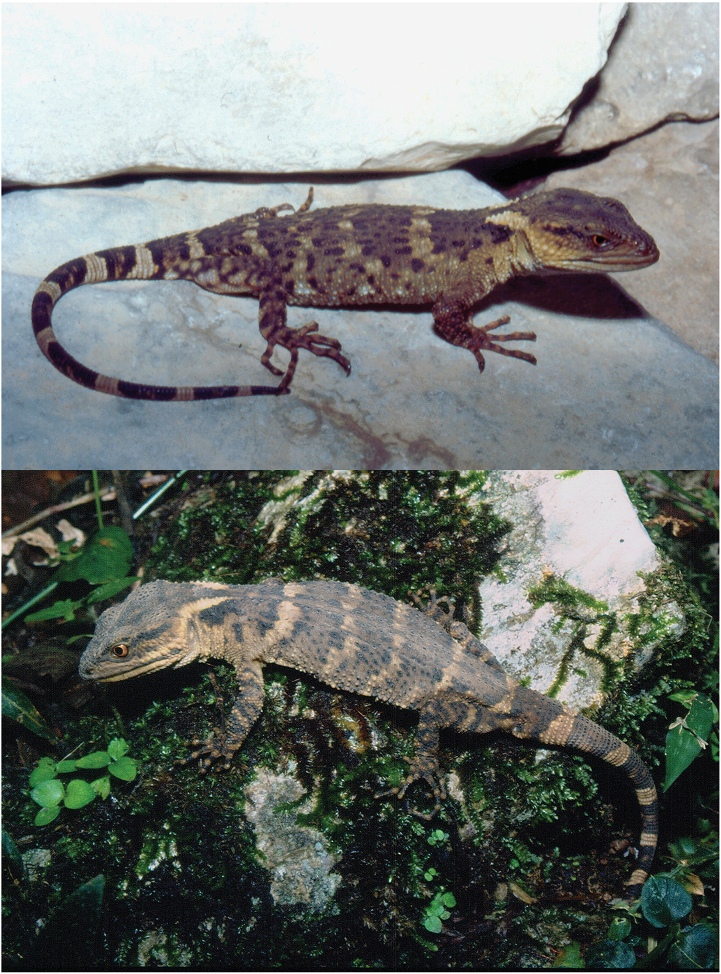
*Xenosaurus fractus* in life. Specimens not collected. Top: Puebla, Municipality of Xochitlán, 200 m N ex-hacienda Apulco, cloud forest, 1450 m. Bottom: Municipality of Huehuetla (no further data). Photographs by Luis Canseco Márquez.

**Figure 4.**
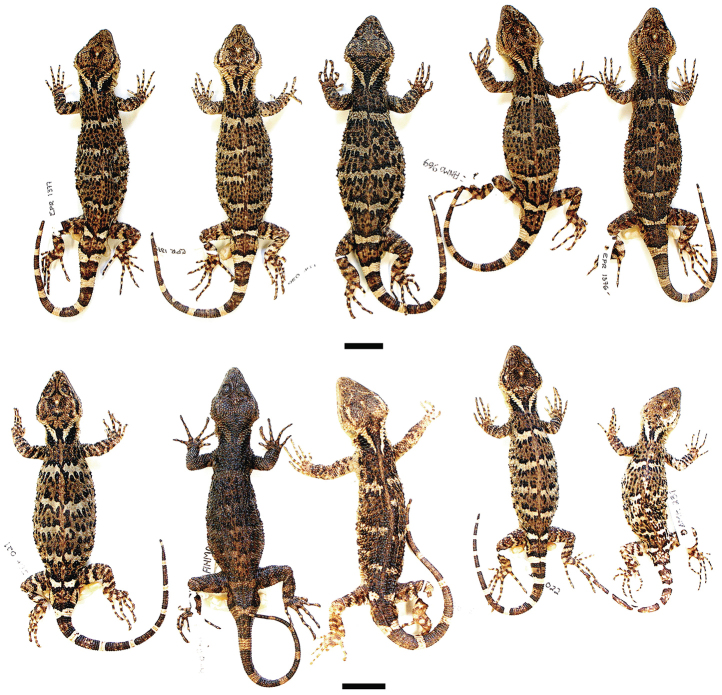
Variation in color pattern (dorsal view) in the type series of *Xenosaurus fractus*. Top row, left to right: MZFC 32181, SVL = 110.4 mm; MZFC 32179, holotype, SVL = 115.3 mm; MZFC 32182, SVL = 122.9 mm; MZFC 32187, SVL = 111.6 mm; MZFC 32180, SVL = 115.1 mm; bottom row, left to right: MZFC 32183, SVL = 105.0 mm; MZFC 32186, SVL = 103.6 mm; MZFC 9579, SVL = 111.4 mm; MZFC 32184, SVL = 94.2 mm; MZFC 32185, SVL = 84.8 mm. Scale bars: 20 mm.

Color pattern (Figs 3–4). Although some photographs of *Xenosaurus fractus* in life are available (Fig. 3), the photographed specimens were not collected. Variation in color pattern in preservative is described below on the basis of eight of the paratypes (Fig. 4; pattern poorly preserved in MZFC 32185). Dorsal background color of head and nape pale brown to dark brown; color pattern similar to that of holotype in all specimens except for dark brown stripes on canthi temporales fairly continuous with black edges of funnel-shaped mark on nape in three specimens (MZFC 32181–82, 32184); and from narrowly to widely separated from latter edges in remaining five; dark brown spot at posteroventral corner of temporal area (near ear) present in three specimens (MZFC 32182–84), absent in remaining five ones; and dark horizontal bar on mid temporal region in one specimen (MZFC 32182).

Four to six dark crossbands between levels of anterior insertion of arms and posterior insertion of legs; crossbands mostly distinct from each other, yet often partially fused with each other and/or misaligned on left and right sides: second and third crossbands misaligned on left and right sides in one specimen (MZFC 9579); second and third crossbands partially fused with each other on right side in two specimens (MZFC 32181–82) and on both sides in one (MZFC 32186); third and fourth crossbands misaligned on left and right sides and partially fused on left side in one specimen (MZFC 32187); all crossbands narrowly connected along midline in four specimens (MZFC 9579, 32180, 32183–84) and additionally second, third, and fourth crossbands partially fused with each other on right side in one specimen (MZFC 32180) and on lower half of both flanks in another one (MZFC 32183).

Dorsal pattern on limbs similar to that of holotype in all specimens, except for dark-edged, cream transverse bar on distal half of thigh absent. Ventral pattern similar to that of holotype in all specimens. Tail with 8–10 dark rings (x = 8.9). Tail pattern in all paratypes similar to that of holotype, except pale midventral spots absent in all rings in two specimens (MZFC 32181, 32187).

Measurements. Females (*n* = 3, SVL = 111.37–122.87 mm): Head length/SVL ratio = 24.2–26.1, x = 25.3; head maximum width/SVL ratio = 20.8–21.1, x = 21.0; head maximum height/head length ratio = 46.2–49.2, x = 47.4; snout length/head length ratio = 35.0–35.9, x = 35.3; shank length/SVL ratio = 14.0–14.9, x = 14.5; tail length/SVL ratio = 92.4–95.4, x = 94.3. Males (*n* = 5, SVL = 94.21–115.05 mm): Head length/SVL ratio = 24.2–26.1, x = 24.9; head maximum width/SVL ratio = 19.5–21.4, x = 20.6; head maximum height/head length ratio = 44.1–50.6, x = 47.5; snout length/head length ratio = 34.0–37.1, x = 35.7; shank length/SVL ratio = 13.2–15.9, x = 14.3; tail length/SVL ratio = 93.3–101.9, x = 98.6.

#### Etymology.

The specific epithet is an adjective in the nominative case (masculine, singular declension) derived from the Latin verb *frangō* (“to break”), meaning “broken” or “fragmented.” The name is in reference to the broken dark crossband on the nape in this species. A continuous dark crossband on the nape, or collar, is present in most species of *Xenosaurus*, and represented in the new species only by black stripes on the sides of the nape, widely separated by posterior extensions of the dark lines on the canthi temporales and the cream subocular stripes.

#### Distribution and ecology.

**Figure 5.**
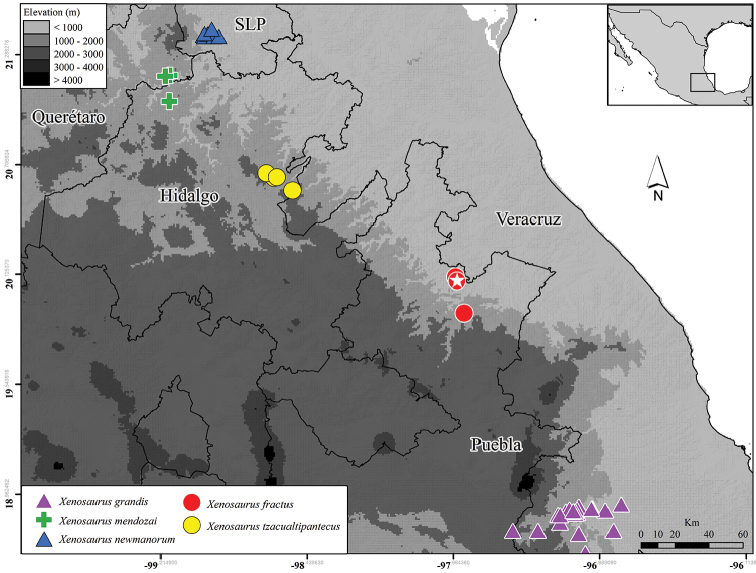
Geographic distribution of *Xenosaurus fractus* (star = type locality) and geographically closest species. Digital elevation model taken from INEGI (2017).

**Figure 6.**
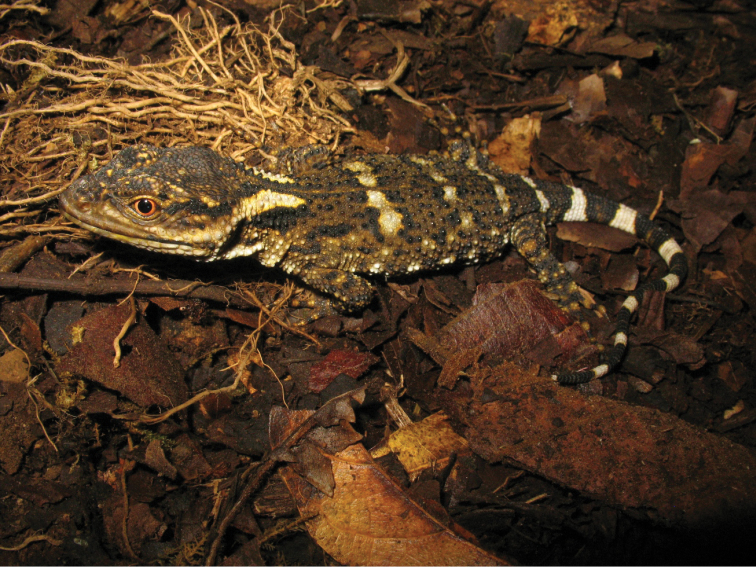
*Xenosaurus* sp from “Zacapoaxtla” (no further data). Specimen not collected. Photograph by Luis Canseco Márquez.

*Xenosaurus fractus* is known to occur in the Municipalities of Huehuetla and Xochitlán on the Caribbean slopes of the Sierra Madre Oriental in northern Puebla (the Sierra Norte de Puebla, Fig. 5). Nine out of the 10 specimens of the type series were collected at or near the towns of Huehuetla or Chilocoyo del Carmen at elevations between approximately 850 and 1000 m, in the crevices of a rocky outcrop between coffee plantations and patches of cloud forest. The original vegetation of the zone is cloud forest. The remaining specimen was collected approximately 18 km SSE of Chilocoyo del Carmen (and approximately 5.6 km SSE Xochitlán, MZFC 32186), but at 1470 m of elevation, in the banks of the Apulco river. A photograph of an uncollected specimen of *Xenosaurus* from “Zacapoaxtla” (Fig. 6), a locality only ca. 5 km south of the latter record at approximately 1800 m of elevation, strongly suggests that the distribution of *X. fractus* extends somewhat further south. Similarly, a recent record of “*Xenosaurus tzacualtipantecus*” from “Tepehican, 2 km N of Tlatlauquitepec (19.0852°N, 97.5068°W; WGS 84), 1930 m elev.” in the Municipality of Tlatlauquitepec, Puebla, by Woolrich-Piña et al. (2016) most probably represents *X. fractus*. Tepehican is actually ca. 1.9 km WNW Tlatlauquitepec (thus coordinates for the “*X. tzacualtipantecus*” record are inaccurate), and approximately 12.1 km SE from the closest definite record of *X. fractus* (approximately 5.6 km SSE Xochitlán, MZFC 32186). Thus, *X. fractus* occurs from approximately 850 m to 1470 m of elevation. However, if the Tepehican record actually corresponds to this species, its upper elevational limit would be higher (1930 m).

## Discussion

On the basis of analyses of ddRADseq data for the genus *Xenosaurus* with a variety of species delimitation methods, Nieto-Montes de Oca et al. (2017) suggested that populations from the Huehuetla region represent a distinct, evolutionary independent (yet undescribed) lineage of *Xenosaurus*. In this study, we documented that populations of *Xenosaurus* from the Huehuetla region also are morphologically diagnosable from all other described species of the genus, including *X. tzacualtipantecus*, with which they have been likely confused (see above). Thus, we corroborated the distinctness of the populations from the Huehuetla region, and provided additional justification for their recognition as a new species (*X. fractus*).

King and Thompson (1968) found a considerable morphological and distributional gap between the species of *Xenosaurus* from northeastern Mexico (*X. newmanorum* and *X. platyceps*) and from southern and southeastern Mexico (*X. agrenon*, *X. arboreus*, *X. grandis*, *X. rackhami*, and *X. sanmartinensis*) recognized at the time (the latter five as subspecies of *X. grandis*). This large gap remained despite the subsequent description of *X. mendozai* (Nieto-Montes de Oca et al. 2013), a species from northeastern Querétaro and adjacent Hidalgo most similar to *X. platyceps*.

The discovery of *X. fractus* and *X. tzacualtipantecus*, from the Sierra Madre Oriental of Puebla and Hidalgo, respectively, has filled the above geographical gap. In addition, the closest species to *X. fractus* is precisely *X. tzacualtipantecus*, from extreme east-central Hidalgo. Excluding the doubtful record of “*X. tzacualtipantecus*” from Puebla (see above), the type-locality of this species is approximately 115.5 km (straight line) WNW from the northernmost record of *X. fractus*. Woolrich-Piña et al. (2012) also reported *X. tzacualtipantecus* from “La Selva, Veracruz, 7.2 km at (sic) E of Huayacocotla.” This locality is approximately 93.7 km WNW from the northernmost record of *X. fractus*; however, La Selva is actually ca. 4.2 km NW, not 7.2 km E, of Huayacocotla, or approximately 102.8 km WNW from the northernmost record of *X. fractus*. At any rate, the area between the Huehuetla region and the closest records of *X. tzacualtipantecus* has not been adequately surveyed. The second closest species to *X. fractus* is *X. grandis* from west-central Veracruz (type-locality approximately 135 km SSE [straight line] from the southernmost definite record of *X. fractus*).

Nieto-Montes de Oca et al. (2017, Fig. 2) showed a record for *X. fractus* from 5 km NE Teziutlán on road to Tlapacoyan, which corresponded to a specimen at the (James Ford) Bell Museum of Natural History (JFBM 2682). This locality is approximately 30 km east from the southernmost record for this species listed herein (approximately 5.6 km SSE Xochitlán, MZFC 32186). However, we were not able to see the Teziutlán specimen. Thus, although we agree that it most likely represents *X. fractus*, we prefer not to refer it to this species for the time being.

Nieto-Montes de Oca et al. (2017) found strong support for a sister species relationship between *X. fractus* and *X. tzacualtipantecus*, a relationship congruent with their close geographic distribution in the Sierra Madre Oriental (see above) and their similar external morphology. For instance, these two species are the only ones in which the broad, cream subocular stripes extend posteriorly along the nape to the first dark crossband on the body, fragmenting the dark crossband on the nape or collar. Morphological data for this relatively new clade of *Xenosaurus* may provide valuable insights into the evolution of morphological, ecological, or other patterns in the genus.

Physiographically, the Sierra Madre Oriental Province consists of folded ridges and intermontane elongated valleys and plateaus; the ridges are more closely spaced in the eastern portion of the province (or Closely Spaced Ridges Subprovince; Ferrusquía-Villafranca 1993), where both *X. fractus* and *X. tzacualtipantecus* are distributed. Nonetheless, it should be noted that *X. fractus* is distributed at lower elevations (850–1470 m [likely 1930 m, see above]) than *X. tzacualtipantecus* (1900–2000 m; Woolrich-Piña et al. 2012), and that the original habitat for *X. fractus* is pure cloud forest, whereas the habitat of *X. tzacualtipantecus* has been described as composed of “cloud forest and pines” (Camarillo-Rangel 1990), conifer forest and cloud forest (Camarillo-Rangel 1998), and “rain forest” (Woolrich-Piña et al. 2012) at its type-locality, and as cloud forest at La Selva (Camarillo-Rangel 1998). *Xenosaurus fractus* is only known from cloud forest, a type of vegetation with a considerably fragmented distribution and that covers only approximately 0.93 % of the Mexican territory: 0.44 % in primary condition and 0.49 % in secondary condition (Challenger and Soberón 2008). Unfortunately, the current extension of this type of vegetation is only approximately one half of its original extension (Challenger and Soberón 2008). Because of this reduction, cloud forest has been considered as one of the most threatened ecosystems in Mexico (Flores-Villela and Gerez 1994). Evidently, species restricted to this habitat require immediate legal protection from the federal and state governments.

### Key to the species of *Xenosaurus*

The following key is based on the key by King and Thompson (1968) and additional relevant literature (Smith and Iverson 1993; Pérez-Ramos et al. 2000; Nieto-Montes de Oca et al. 2001, 2013; and Woolrich-Piña et al. 2012).

**Table:** 

1	Dark crossband on the nape or collar usually continuous, enclosed anteriorly by the pale subocular stripes, which extend medially onto the nape to produce a light crossband (often interrupted medially) similar to those on the trunk, and posteriorly by a pale crossband in the scapular region separating it from the first dark crossband on the trunk	**2**
–	Dark crossband on the nape or collar divided longitudinally by the subocular stripes, which extend posteriorly along the nape to the first dark crossband on the trunk, and no light crossband in the scapular region separating the fragments of the dark crossband on the nape from the first dark crossband on the trunk	**12**
2	Usually 2–3 enlarged, rounded supraoculars forming a longitudinal row; canthus temporalis lacking; venter usually uniform light gray to white in color, occasionally with few dark specks on the sides	**3**
–	A longitudinal row of 3–5 enlarged hexagonal supraoculars that are wider than long; canthus temporalis usually present, consisting of a longitudinal series of enlarged scales that are distinct from the smaller granular scales of the temporal region; venter variable	**5**
3	Tail length/SVL 0.76–0.90; a medial postrostral usually present	***X. mendozai***
–	Tail length/SVL 0.92–1.13; a medial postrostral usually not present	**4**
4	Head narrowly triangular, 0.75–0.83 times as wide as long; thick, 0.63–0.68 times as high as wide; transverse rows of scales between axilla and groin 33–37; scales per row at widest part of belly, 17–18; supraorbital semicircles in contact, not separated by a median scale row; tympanum bare, without a thin, scaled membrane; light crossbands on body not continuous across the midline; dark V-shaped nape blotch, attenuate and pointed posteriorly	***X. newmanorum***
–	Head broadly triangular, 0.86–0.97 times as wide as long; flat, 0.47–0.54 times as high as wide; transverse scale rows between axilla and groin 40–42; scales per row at widest part of belly, 20–23; supraorbital semicircles separated by a single, median row of scales; tympanum covered by a thin, scaled membrane; transverse light bands on body consisting of the ground color, continuous dorsally, and accentuated by enamel-white tubercles; dark nape blotch W-shaped, truncate posteriorly	***X. platyceps***
5	2–6 (usually 3–4) white spots, sometimes faint, on the infralabial-labiomental region; second pair of chinshields usually in medial contact with each other; tail length/SVL 0.73–0.79; venter uniform light gray or with few, small dark spots on the sides	***X. phalaroanthereon***
–	White spots on the infralabial-labiomental region absent; second pair of chinshields usually separated from each other; tail length/SVL 0.78–1.10; venter variable	**6**
6	Maximum count of longitudinal ventral scale rows 25–29; postparietal dark spots usually present; 19–22 subdigital lamellae on fourth toe; postocular and zygomatic ridges in contact; venter immaculate	***X. rectocollaris***
–	Maximum count of longitudinal ventral scale rows 17–24; postparietal dark spots absent; 23–31 subdigital lamellae on fourth toe or, if subdigital lamellae on fourth toe < 23 or venter uniform light gray, postocular and zygomatic ridges separate; venter variable	**7**
7	Postrostral single, postrostral and nasal scales in contact; supraorbital semicircles usually separated by two scales; labiomentals usually reaching the first chinshield	***X. penai***
–	Postrostral fragmented or divided, postrostral and nasal scales separated; supraorbital semicircles usually narrowly separated by a single scale; labiomentals usually reaching the second or third chinshield	**8**
8	Head broadly triangular, 0.79–0.96 times as wide as long	**9**
–	Head narrowly triangular, 0.73–0.83 times as wide as long	**11**
9	Tail 0.86–1.10 times snout-vent length; pattern of lateral tubercles obscured by small folds and creases in skin radiating from lateral fold; chest scales arranged in reticulating series of short rows or randomly arranged; venter light-colored with dark spots that may form distinct ventrolateral bars	***X. grandis***
–	Tail 0.79–0.97 times snout-vent length; lateral tubercles arranged in oblique longitudinal series; chest scales arranged in oblique transverse series; venter uniform light gray in color, or with dark bars	**10**
10	Dorsal ground color very dark, transverse light bands reduced even to obsolete paravertebral light spots, and with light tubercles arranged in transverse series; dark nape blotch obscured by general dark coloration, but V-shaped and pointed posteriorly; venter uniform light gray in color	***X. arboreus***
–	Dorsal ground color medium to dark brown, transverse light bands usually present, but may be reduced to row of spots; dark nape blotch W-shaped and rounded posteriorly; venter usually with dark bars, which may be reduced or absent	***X. agrenon***
11	Transverse light bands on body frequently no lighter than ground color; dark markings reduced to spots and blotches	***X. rackhami***
–	Transverse light bands on body always lighter than ground color; dark bands irregular, but continuous across dorsal surface	***X. sanmartinensis***
12	Black-edged, cream transverse bars on mid upper arm, forearm, thigh, and shank usually present; 26–34 subdigital lamellae on the fourth toe	***X. fractus***
–	Black-edged, cream transverse bars on mid upper arm, forearm, thigh, and shank usually absent; 23–28 subdigital lamellae on the fourth toe	***X. tzacualtipantecus***

## Supplementary Material

XML Treatment for Xenosaurus fractus

## Supplementary materials

Supplementary material 1 Specimens examined Data type: specimens data This is an open access article distributed under the terms of the Creative Commons Attribution License (CC BY 4.0), which permits unrestricted use, distribution, and reproduction in any medium, provided the original author and source are credited.Nieto-Montes de Oca A, Sánchez-Vega H, Durán-Fuentes I

Supplementary material 2 Variation in selected characters in the genus *Xenosaurus* Data type: species data Explanation note: *Xenosaurus* clades after Nieto-Montes de Oca et al. (2017). This is an open access article distributed under the terms of the Creative Commons Attribution License (CC BY 4.0), which permits unrestricted use, distribution, and reproduction in any medium, provided the original author and source are credited.Nieto-Montes de Oca A, Sánchez-Vega H, Durán-Fuentes I
