# Supplementary material for: A new species of knob-scaled lizard (Xenosauridae, Xenosaurus) from the Sierra Madre Oriental of Puebla, Mexico
Source: Zookeys. 2018 Feb 15;(737):141–60. doi: 10.3897/zookeys.737.15095 (PMC5904495; doi:10.3897/zookeys.737.15095)
Supplement: Supplementary material 2 — Variation in selected characters in the genus Xenosaurus [file zookeys-737-141-s002.pdf]

Table 2.—Variation in selected characters in the genus *Xenosaurus*. *Xenosaurus* clades after Nieto-Montes de Oca et al. (2017).

	newmanorum clade			*tzacualtipantecus* clade		*grandis* clade					*rackhami* clade		
Characters/taxon	*mendozai* $n = 26$	*newmanorum* $n = 11$	*platyceps* $n = 13$	*fractus* $n = 10$	*tzacualtipantecus* $n = 8$	*agrenon* $n = 14$	*grandis* $n = 14$	*penai* $n = 7$	*phalaroanthereon* $n = 16$	*rectocollaris* $n = 11$	*arboreus* $n = 5$	*rackhami*[1] $n = 15$	*sanmartinensis* $n = 16$
Medial postrostral present	Rarely (15.4%)	Usually (90.9%)	Usually (92.3%)	Yes	Usually (71.4%)	Rarely (21.4%)	Yes	Yes	Yes	Yes	Yes	Yes	Yes
Postrostrals on each side of medial postrostral/midline	2	2–3	2–3	Usually 1 (95.0%)	1	0–1	Usually 1 (92.9%)	0–1	0–1	Usually 0–1 (90.9%)	1	1	Usually 0–1 (96.9%)
Postocular and zygomatic ridges	Separate	Separate	Separate	Variable	In contact	Separate	Separate	Separate	In contact	In contact	Separate	In contact	In contact
Canthus temporalis	Absent	Absent	Absent	Weak	Weak	Weak	Weak	Well developed	Absent	Absent	Weak	Well developed	Well developed
White spots on infralabial–labiomental region	Absent	Absent	Absent	Absent	Absent	Absent	Absent	Absent	Present	Absent	Absent	Absent	Absent
Second chinshields in medial contact	Never	Never	Never	Never	Never	Usually (64%)	Never	Never	Usually (94%)	Never	Never	Rarely (7%)	Never
Subdigital lamellae on fourth toe	$x = 24.3$ (23–26)	$x = 30.3$ (29–32)	$x = 26.0$ (23–28)	$x = 29.9$ (26–34)	$x = 25.6$ (23–28)	$x = 25.5$ (23–28)	$x = 27.5$ (24–29)	$x = 25.0$ (24–27)	$x = 19.4$ (19–22)	$x = 20.5$ (20–22)	$x = 24.4$ (23–26)	$x = 28.2$ (25–31)	$x = 28.4$ (27–30)
Tail length/snout-vent length[2]	0.76–0.90	0.93–1.03	0.92–1.13	0.93–1.02	0.87–1.00	0.78–0.97	0.86–1.10	0.84–1.04	0.73–0.79	0.89–1.06	0.81–0.88	0.87–1.09	0.88–1.03
Collar fragmented longitudinally by the posterior extensions of the subocular stripes	No	No	No	Yes	Yes	No	No	No	No	No	No	No	No
Venter	Usually with few, scattered dark specks on sides	Usually immaculate or with few, small dark spots on sides (81.8%)	Usually immaculate or with few, small dark spots on sides (92.3%)	With dark transverse bars	Usually with numerous small, dark scattered spots	With dark transverse bars	With dark transverse bars	With dark transverse bars	Immaculate (69%) or with few, small dark spots on sides	Immaculate	Uniform pale gray	With dark transverse bars	Usually with dark transverse bars (93.8%)

[1] Sample size for subdigital lamellae on the fourth toe = 14.

[2] Data for *X. agrenon*, *X. arboreus*, *X. grandis*, *X. newmanorum*, *X. platyceps*, *X. rackhami*, and *X. sanmartinensis* taken from King and Thompson (1968). Sample sizes not specified.
